# Nitric Oxide on Extracorporeal Membrane Oxygenation in Neonates and Children (NECTAR Trial): Protocol for a Randomized Controlled Trial

**DOI:** 10.2196/43760

**Published:** 2023-03-15

**Authors:** Adrian C Mattke, Kerry Johnson, Kristen Gibbons, Debbie Long, Jeremy Robertson, Prem S Venugopal, Antje Blumenthal, Andreas Schibler, Luregn Schlapbach

**Affiliations:** 1 Paediatric Intensive Care Unit Children's Health Queensland Queensland Children's Hospital South Brisbane Australia; 2 School of Medicine University of Queensland Herston Australia; 3 Child Health Research Centre The University of Queensland South Brisbane Australia; 4 School of Nursing Centre for Healthcare Transformation Queensland University of Technology Brisbane Australia; 5 Paediatric Haematology and Haemophilia Service Queensland Children's Hospital South Brisbane Australia; 6 Department for Cardiac Surgery Queensland Children's Hospital Children's Health Queensland South Brisbane Australia; 7 Frazer Institute The University of Queensland Brisbane Australia; 8 Wesley Medical Research Institute Brisbane Australia; 9 University Children's Hospital Zurich University of Zurich Zurich Switzerland

**Keywords:** extracorporeal membrane oxygenation, randomized controlled trial, children, nitric oxide, child, RCT, ECMO, neonate, neonatal, infant, baby, babies, life support, mortality, pulmonary, cardiovascular, heart, lung

## Abstract

**Background:**

Extracorporeal membrane oxygenation (ECMO) provides support for the pulmonary or cardiovascular function of children in whom the predicted mortality risk remains very high. The inevitable host inflammatory response and activation of the coagulation cascade due to the extracorporeal circuit contribute to additional morbidity and mortality in these patients. Mixing nitric oxide (NO) into the sweep gas of ECMO circuits may reduce the inflammatory and coagulation cascade activation during ECMO support.

**Objective:**

The purpose of this study is to test the feasibility and safety of mixing NO into the sweep gas of ECMO systems and assess its effect on inflammation and coagulation system activation through a pilot randomized controlled trial.

**Methods:**

The Nitric Oxide on Extracorporeal Membrane Oxygenation in Neonates and Children (NECTAR) trial is an open-label, parallel-group, pilot randomized controlled trial to be conducted at a single center. Fifty patients who require ECMO support will be randomly assigned to receive either NO mixed into the sweep gas of the ECMO system at 20 ppm for the duration of ECMO or standard care (no NO) in a 1:1 ratio, with stratification by support type (veno-venous vs veno-arterial ECMO).

**Results:**

Outcome measures will focus on feasibility (recruitment rate and consent rate, and successful inflammatory marker measurements), the safety of the intervention (oxygenation and carbon dioxide control within defined parameters and methemoglobin levels), and proxy markers of efficacy (assessment of cytokines, chemokines, and coagulation factors to assess the impact of NO on host inflammation and coagulation cascade activation, clotting of ECMO components, including computer tomography scanning of oxygenators for clot assessments), bleeding complications, as well as total blood product use. Survival without ECMO and the length of stay in the pediatric intensive care unit (PICU) are clinically relevant efficacy outcomes. Long-term outcomes include neurodevelopmental assessments (Ages and Stages Questionnaire, Strength and Difficulties Questionnaire, and others) and quality of life (Pediatric Quality of Life Inventory and others) measured at 6 and 12 months post ECMO cannulation. Analyses will be conducted on an intention-to-treat basis.

**Conclusions:**

The NECTAR study investigates the safety and feasibility of NO as a drug intervention during extracorporeal life support and explores its efficacy. The study will investigate whether morbidity and mortality in patients treated with ECMO can be improved with NO. The intervention targets adverse outcomes in patients who are supported by ECMO and who have high expected mortality and morbidity. The study will be one of the largest randomized controlled trials performed among pediatric patients supported by ECMO.

**Trial Registration:**

Australian New Zealand Clinical Trials Registry ACTRN12619001518156; https://www.anzctr.org.au/Trial/Registration/TrialReview.aspx?id=376869

**International Registered Report Identifier (IRRID):**

DERR1-10.2196/43760

## Introduction

Extracorporeal membrane oxygenation (ECMO) has gained widespread acceptance as a rescue treatment for refractory cardiovascular and respiratory failure among neonates, children, and adults [1-3]. While morbidity and mortality in ECMO are predominantly affected by the severity of preexisting disease, the contribution of iatrogenic factors to patient-centered outcomes is becoming increasingly recognized [4]. Despite major improvements in ECMO devices, the exposure of host blood to large artificial organ surfaces, mechanical cell stress, and reperfusion injury result in the activation of the coagulation system and generate a severe inflammatory response [5]. Studies on extracorporeal circuits (including bypass circuits) have shown that hypoxic-ischemic injury and the release of damage-associated molecular patterns trigger an inflammatory cascade closely related to sepsis-induced systemic inflammatory response syndrome. It is characterized by endotoxin release, leukocyte and complement activation, and widespread activation of inflammatory mediators, resulting in endothelial leak, increased oxygen consumption, and organ dysfunction [6]. Both intrinsic factors and extrinsic factors, such as exposure to injured tissue, contribute to excessive inflammation and coagulation system activation and may be further aggravated by cross talk across the complement and coagulation systems [7]. Therefore, it is not surprising that thrombosis and hemorrhage remain among the most common complications seen with ECMO [8].

These ECMO-related side effects expose patients to substantial morbidity and can lead to death or devastating long-term outcomes, even if the primary reason for requiring ECMO has been resolved. Importantly, some pediatric data show that more than 85% of patients who die during or after their ECMO run have developed either bleeding or clotting complications [9].

Nitric oxide (NO) mediates anti-inflammatory host responses and regulates both endothelial functions as well as microvascular inflammation. The mixing of NO into the sweep gas that runs into the oxygenator in neonates and children undergoing cardiopulmonary bypass has been investigated in several pilot studies with promising results, and a large randomized controlled trial (RCT) has recently been published [10-13]. In this trial, the effects of NO used in a bypass circuit for pediatric cardiac surgery were tested [13]. No effect on mortality or on the duration of ventilation was observed, though the safety of NO was high. The treatment duration on cardiopulmonary bypass is a few hours, whereas ECMO support lasts days to weeks, making the 2 clinical scenarios not directly comparable. In addition, mixing NO into the sweep gas (at 30, 50, and 80 ppm) in an animal model of ECMO significantly reduced the host response, with suppression of tumor necrosis factor *α* (TNF-*α*) and interleukin (IL)-6 expression (Fraser J, unpublished data, April 2023). A single-center observational study using historical controls reported the safety and outcomes of NO at 20 ppm mixed into the sweep gas of the ECMO oxygenator. The authors observed a lower blood product administration and a marginal improvement in the survival of patients on NO compared to the historical controls without any NO. There are no randomized studies on the effect of NO mixed into the oxygenator on patients treated with ECMO.

The Nitric Oxide on Extracorporeal Membrane Oxygenation in Neonates and Children (NECTAR) study was designed to investigate in a randomized clinical trial whether mixing NO into the sweep gas in neonates and children treated with ECMO is feasible (recruitment, randomization, and protocol compliance) and safe (accurate NO delivery and methemoglobin levels). Efficacy will be tested by measuring ECMO-free days (the number of days that patients spend alive and free from ECMO is censored at 30 and 90 days) and by assessing the inflammatory host response and coagulation activation. We hereby describe the NECTAR trial protocol.

## Methods

The NECTAR trial is an investigator-initiated, 50-patient, open-label, single-center, pilot RCT with neonates and children who require ECMO support.

### Study Setting

A tertiary pediatric intensive care unit (PICU) in Australia with approximately 30 ECMO runs annually.

### Participants

Eligible children will be identified during the clinical deterioration typically seen prior to ECMO support commencement, such as progressive respiratory, cardiovascular, and cardiorespiratory failure, including cardiopulmonary arrest. If the situation permits, prospective consent will be sought; otherwise, delayed consent or consent to continue the approach will be applied.

### Inclusion Criteria

All infants and children (birth to ≤16 years) that are commenced on ECMO will be eligible for inclusion in the study if there is the ability to obtain prospective consent or consent to continue from parents or guardians. In addition, infants or children that are commenced on ECMO at the colocated Neonatal Intensive Care Unit will be eligible for inclusion in the study. Patients who are commenced on ECMO outside of these units will not be eligible for the study.

### Exclusion Criteria

Neonates and children treated with ECMO who were retrieved to the PICU on ECMO, those with preexisting methemoglobinemia (methemoglobin [MetHb]>3%), and patients managed on a ventricular assist device only without an oxygenator were present in the ECMO circuit.

### Randomization and Blinding

Treatment group allocation will be performed using a web-based REDCap electronic data capture tool hosted by The University of Queensland [14,15]. A randomization sequence using variable block randomization with a 1:1 ratio was uploaded into REDCap prior to the screening of the first patient. Randomization is stratified by veno-arterial (VA) versus veno-venous (VV) support. Where conversion of VV to VA or VA to VV occurs, patients will remain in the initial randomization allocation and strata. When a second ECMO run is commenced, the patient will be randomized again.

### Consent

Prospective consent will be sought from the parents or guardians of any child for whom a decision to be commenced on ECMO has been made. Where possible, this consent will be obtained before the commencement of ECMO. However, due to parental stress and cognitive overload at the time of cannulation, consent can also be obtained following randomization under the consent to continue model (termed “delayed consent” previously) [16]. Prior to or after ECMO commencement, parents will be provided with study information, including printed study flyers and links to web-based study documentation.

### Blinding of the Intervention

Blinding is not possible, as the NO delivery device makes a distinctive noise during standard operation. Further, the NO levels need to be adjusted depending on sweep flow, which requires nurses by the side to identify whether NO is being delivered or not.

### Interventions

Infants and children will be randomly assigned to receive either NO or standard care. Those allocated to the intervention (NO) will receive 20 ppm of NO mixed into the sweep gas running into the oxygenator during their ECMO run. The NO concentration will be maintained at 20 ppm using a NO delivery system (Sokinox, Maquet), or a similar device. Continuous sampling of NO and NO_2_ concentrations is performed by the Sokinox machine by gas aspiration after the NO injection site. Patients allocated to the standard study arm will receive the standard oxygen-air mix in the ECMO oxygenator as per institutional practice and protocol.

### Relevant Concomitant Coagulation Management and Intensive Care Treatment

Apart from mixing NO into the sweep gas flow of the ECMO system, no other patient management parameters will be changed, and institutional practices and protocols will be followed. Methods and goals of anticoagulation will remain unchanged for any other ECMO management and treatments, such as decisions to decannulate, ventilation support, inotrope management, fluid intake, fluid removal practices, or antimicrobial or immunological treatments.

### Safety Data Monitoring

A data and safety monitoring board (DSMB) will monitor the conduct of the study and the accrual of data. The DSMB will consist of a chairperson (experienced in clinical trials), a pediatric intensivist, a cardiac surgeon, and a statistician. The DSMB will be independent of the study investigators and will monitor adverse events in the study as well as ensure the validity and timeliness of the data collection. The DSMB may advise the Chief Investigator to terminate the trial, which would necessitate safety concerns in this step. The DSMB is expected to meet at least 4 times during the study period.

### Monitoring

The study team will be responsible for all documentation of the study nurse’s and the investigator’s training records and credentials. All consent forms will be reviewed by the study team. The perfusion data collection forms will be compared to the source documentation to ensure both the completeness and accuracy of the data. All severe adverse events will be recorded and reported to the local Human Research and Ethics Committee (HREC) as per the institutional guidelines.

### Ethics Approval

This protocol and the informed consent document, and any subsequent modifications, have been reviewed and approved by the human research ethics committee (reference number HREC/18/QCHQ/49018). This study will be conducted in compliance with this version of the protocol. Any change to the protocol document or informed consent document that affects the scientific intent, study design, patient safety, or anything that may affect a participant’s willingness to continue in the study is considered an amendment, and therefore will be written and filed as an amendment to this protocol or the informed consent document. All protocol deviations will be reported to the principal investigator, recorded in REDCap, and reported to HREC. Protocol deviations and violations will be assessed for clarification by the principal investigator. The study was approved by the local HREC (HREC/18/QCHQ/49018) and has been registered with the Australian New Zealand Clinical Trials Registry (ACTRN12619001518156). The NECTAR trial commenced recruitment in August 2020

### Sample Size

The NECTAR trial is a pilot RCT with the main focus on feasibility, safety, and exploration of efficacy. A sample size of 50 patients has been chosen in line with the recommendations for pilot studies that anticipate a medium effect size for a continuous primary outcome for the main trial [17].

### Data Collection, Management, and Analysis

#### Data Collection

Baseline variables (demographics, primary diagnosis, comorbidities including syndromes), precannulation disease severity, precannulation surgery (such as cardiac surgery), cannulation technique, primary and secondary end points, as well as predetermined physiological variables of interest, will be recorded and entered prospectively into a purpose-built REDCap database [14,15]. Treatment that is administered continuously (such as heparin or inotropes) will be recorded as maximal values that were maintained for at least 4 hours. Physiological and blood parameters will be recorded precannulation, at 1, 12, and 24 hours post cannulation, and daily thereafter. The duration of circulatory, renal, and ventilatory support on and off ECMO will be recorded. Gross functional performance assessment will be recorded upon admission to the PICU and upon discharge from the hospital [18].

Participant data confidentiality will be ensured by the study coordinator, the research staff, and the institution sponsoring the trial. All data, as well as the study protocol and any other information that this trial may generate, will be held in strict confidence.

#### Biobanking

Blood samples for inflammatory and coagulation pathway activation will be collected prior to cannulation, as well as at 1, 12, and 24 hours post commencement of ECMO. These samples will be frozen using standard operating procedures and batch tested.

## Results

### Study Outcomes

The NECTAR trial is a pilot RCT; hence, it will focus on safety and feasibility outcomes in addition to exploring efficacy outcomes as described in Textbox 1. This work was supported by grants from the Children’s Hospital Foundation, Brisbane, Australia, and by a grant from the Thrombosis and Hemostasis Society of Australia. The results are expected to be published at the end of 2023 or early 2024.

**Main study outcomes and secondary analyses for the Nitric Oxide on Extracorporeal Membrane Oxygenation in Neonates and Children (NECTAR) trial.**

**Main study outcomes**

- Feasibility outcomes
  Monthly recruitment rate
  Randomized to screened patient ratio
  Compliance with the study protocol (with particular focus on the timing of the initiation of nitric oxide (NO) and the maintenance of NO for the duration of the study)
- Safety outcomes
  Failure rate of gas delivery
  Carbon dioxide control of patients
  Methemoglobin levels
- Efficacy outcomes
  Host systemic inflammation levels at 1, 12, and 24 hours
  Coagulation system activation and consumption
  Clotting and bleeding complications
  Rate of clotting and bleeding events

**Secondary outcomes**

- Survival without extracorporeal membrane oxygenation (ECMO), censored at 30 and 90 days post randomization. Patients dying within 30 and 90 days of presentation will be considered as 0 days, to account for the competing effect of mortality on ECMO-free survival.
- Survival free of pediatric intensive care unit (PICU; censored at 30 and 90 days)
- Hospital length of stay post randomization
- Median daily blood product usage
- Complement system activation
- Child neurodevelopment, function, and quality of life at 6 and 12 months, as measured by:
  Ages and Stages Questionnaire (<5 years of age) OR Strength and Difficulties Questionnaire (>5 years of age)
  Pediatric Quality of Life Inventory
  Pediatric Emotional Distress Scale (2-5 years) OR Children’s Revised Impact of Events Scale (>5 years)
  Behavior Rating Index of Executive Functioning – Preschool (2-5 years) OR Behavior Rating Index of Executive Functioning – 2 (>5 years)
  Vineland Adaptive Behavior Scale
- Parent functioning at 6 and 12 months, as measured by:
  Parenting Stress Index – Short Form
  Kessler-6
  Primary Caregiver – Post Traumatic Stress Disorder Scale

### Feasibility Outcomes

The focus will be on the monthly recruitment rate, the randomized to screened patient ratio, and protocol adherence, that is, how many blood collection points, computer tomography scans of oxygenators, and daily indirect oxygenator clot measurements (by extracorporeal life support assessment monitor) were completed.

### Safety Outcomes

There will be a focus on sweep gas delivery failure related to NO administration. Specifically, an arterial oxygen saturation of less than 80 due to a sweep gas interruption related to NO administration or carbon dioxide control outside of 30 to 50 mm Hg due to NO administration will be recorded as a safety event. The methemoglobin (MetHb) levels during the time of ECMO will be measured, and events above 3% related to NO administration will be noted.

### Efficacy Outcomes

Host inflammation and complement components will be assessed before cannulation and at 1, 12, and 24 hours post cannulation by determining serum concentrations of C-reactive protein, procalcitonin, IL-1b, IL-6, IL-8, IL-10, and TNF-*α*. At the same time points, coagulation system activation will be assessed by measuring the international normalized ratio (INR), prothrombin time (PT), activated partial thromboplastin time (APTT), thromboelastography (TEG), antithrombin, soluble CD40L, P-selectin, platelet factor 4 levels, and ADAMTS13 release (see Textbox 2). A single measurement of Von Willebrand factor at initiation followed by daily measurements will be performed. After the initial 24 hours, further daily laboratory testing will include D-Dimers, TEG, INR, PT, APTT, anti Xa and activated clotting time (by TEG), and plasma-free hemoglobin. Clotting and bleeding complications and the rate of clotting and bleeding events will be measured. Clotting events are defined as the need to change ECMO circuit componentry due to high transmembrane pressures, high D-Dimers, or high free plasma hemoglobin. Bleeding rates will be measured by both the amount of bleeding per day as well as the daily requirement for blood product administration.

**Coagulation, inflammation, and complement system measurements.**

Coagulation system measurements

- Change in granule (soluble CD40 ligand and P-selectin) and chemokine (platelet factor 4) levels released by activated platelets
- ADAMTS13 release
- Change in van Willebrand factor concentrations during and after the extracorporeal membrane oxygenation (ECMO) run

Changes in inflammatory and complement system activation

- Change in C-reactive protein, procalcitonin, interleukin (IL)-1b, IL-6, IL-8, IL-10, and tumor necrosis factor *α* (inflammatory mediators) levels; Complement (C)1q, Complement Factor B, Complement Factor H, C5a, C9, and Mannose-Binding Lectin (classic and alternate complement pathways); and C3, C3 and CH50.

### Secondary Clinical Outcomes

The secondary clinical outcomes include mortality at 30 and 90 days. ECMO-free and PICU-free survival censored at 30 and 90 days post randomization, length of stay at the hospital post randomization, average daily blood product usage, complement system activation with measurements of complement (C)1q, C5a, C9, C3, C4, complement factor B, complement factor H, and Mannose-binding Lectin, as well as neurodevelopmental function and quality of life at 6 and 12 months, will be considered (Textbox 3).

**Subanalyses for the Nitric Oxide on Extracorporeal Membrane Oxygenation in Neonates and Children (NECTAR) trial.**

- Cardiac, respiratory, and extracorporeal membrane oxygenation–assisted cardiopulmonary resuscitation outcomes (proportions alive at 30 and 90 days for each group)
- Circuit duration (time to decannulation or time to circuit change) per treatment group
- Renal function measured as creatinine and urine output changes during extracorporeal membrane oxygenation (ECMO) per treatment group
- Renal replacement treatment on ECMO (type of renal replacement therapy, duration of slow continuous ultrafiltration, type of filter, and duration of filter viability)
- Incidence of seizures diagnosed during ECMO or up to 48 hours after decannulation
- Transmembrane oxygenator pressure assessment every 24 hours
- Oxygenator clot volume as assessed by the extracorporeal life support assessment monitor
- Oxygenator clot volume (by computer tomography imaging)
- Hemolysis (plasma-free hemoglobin) per treatment group
- Mean daily heparin dose per kg required to meet target anticoagulation per treatment group

### Subanalyses

We will further investigate the oxygenator’s function and structure. The former will be done by postoxygenator blood gas sampling, and the latter by oxygenator clot volume assessment by computer tomography scanning. Outcomes by age group (<28 days, 28 to 365 days, and greater than 365 days) will be analyzed.

### Statistical Analysis Plan

#### Analysis Plan

Descriptive statistics will be used to report the baseline characteristics of the total study cohort and each subgroup. The primary efficacy outcome measure investigating days free of ECMO will be analyzed using quantile regression incorporating treatment group and VA or VV support (stratification variable) as fixed effects, with the treatment group effect estimate and 95% CI reported (assuming the data is abnormally distributed). Exploratory subgroup analysis by cardiac, respiratory, and ECMO-assisted cardiopulmonary resuscitation support will be performed. Analysis of secondary outcomes includes comparisons of measurements and proportions, with confidence intervals of differences as the major method of presentation. The primary analysis will be by intention-to-treat. If appropriate, per-protocol analyses will be performed as well and compared to the intention-to-treat. Statistical significance will be set at the .05 level and no adjustment for multiple comparisons will be made, noting the exploratory nature of the analysis. CIs will be generated using quantile regression for abnormally distributed continuous data, a 2-sided *t* test for normally distributed data, and a test of 2 proportions for binary data.

#### Long-term Outcomes

Neurodevelopmental and functional outcomes (using phone interviews and web-based questionnaires) will be documented at baseline (assessment of premorbid function during PICU admission) using the test battery described in Table 1. Assessments will focus on neurodevelopment, quality of life, executive functioning, adaptive behaviors, functioning, and parental outcomes. Assessments will occur at 6 and 12 months post cannulation. The following will be used for children younger than 5 years (for respective neurodevelopmental assessment areas): (1) ages and stages questionnaire, (2) pediatric emotional distress scale, and (3) behavior rating index of executive functioning for preschoolers. For all other children (and other neurodevelopmental function assessments), the following will be used: (1) strengths and difficulties questionnaire, (2) children’s revised impact of events scale, (3) vineland adaptive behavior scale, (4) pediatric cerebral performance category, (5) pediatric outcome performance category, and (6) pediatric quality of life inventory. For parental outcomes, the Kessler scale, as well as the Primary Carer – Post Traumatic Stress Disorder and Parenting Stress Index – Short Form will be used.

The neurodevelopmental outcomes will be reported separately from the main study.

**Table 1:** Long-term outcome measures at 6 and 12 months after pediatric intensive care unit discharge.

Measure	<5 years of age	≥5 years of age
Neurodevelopment	Ages and Stages Questionnaire (ASQ)	Strengths and Difficulties Questionnaire (SDQ)
Quality of life	Pediatric Quality of Life Inventory	Pediatric Quality of Life Inventory
Distress	Pediatric Emotional Distress Scale (2-4 years) (PEDS)	Children’s Revised Impact of Events Scale (≥5 years) (CRIES)
Executive functioning	Behavior Rating Index of Executive Functioning for Preschoolers (2-4 years) (BRIEF-P)	Behavior Rating Index of Executive Functioning (≥5 years) (BRIEF)
Adaptive behavior	Vineland Adaptive Behavior Scale (VABS)	VABS
Functioning	Functional Status Score (FSS), Pediatric Cerebral Performance Category (PCPC), and Pediatric Outcome Performance Category (POPC)	FSS, PCPC, and POPC
Parent outcomes	Kessler (K6), Primary Carer – Post Traumatic Stress Disorder (PC-PTSD), and Parenting Stress Index – Short Form (PSI-4-SF)	K6, PC-PTSD, and PSI-4-SF

### Adverse Events

Given the severity of the underlying disease leading to the requirement for ECMO, patients are expected to have a high rate of adverse events unrelated to the study intervention. Events that are in the expected range of adverse events during standard treatment will therefore not be reported as related to the study intervention, as previously described [19]. Adverse events that are likely related to the study intervention will be reported. Adverse events likely related to NO delivery during ECMO support, such as failure to deliver sweep gas to the oxygenator, poor patient carbon dioxide control due to sweep gas failure, and increased MetHb (MetHb >3%) at any of the blood gas sampling points, will be reported [11,12]. Death will be reported as an adverse event and will be assessed as to whether it may be related to the study intervention. The study team, together with the treating physician, will assess each patient’s case and make an initial assessment of whether death occurred due to the intervention. The final assessment is to be confirmed with the DSMB.

## Discussion

### Principal Findings

The NECTAR trial will be the first RCT to assess the effect of NO on bleeding, clotting, and inflammatory complications in neonates and children supported on ECMO. It will establish the feasibility and safety of the procedure to allow the design of a trial powered for patient-centered outcomes.

Children requiring ECMO support continue to have a very high mortality risk. Both bleeding and clotting events are significantly associated with mortality and morbidity in this group of patients, and—in combination with inflammatory activation—they continue to pose a challenge to which no therapeutic answer has been found to date. The addition of NO to the sweep gas to reduce bleeding, clotting, and inflammatory complications is a promising therapeutic approach that has so far only been tested in vitro, in historical comparisons, or in RCTs on cardiopulmonary bypass settings rather than ECMO. Given the anticipated high safety of the intervention, the approach has the potential to improve patient-centered short- and long-term outcomes.

### Limitations

The patient group requiring ECMO is very heterogeneous, and due to the pilot study size, achieving a normal distribution of risk factors in the 2 study groups may be unlikely. In addition, this pilot trial is not powered to assess associations between the intervention and clinical outcomes such as ECMO-free survival. It is designed to test the feasibility, safety, and efficacy of such an intervention and the conduct of such a study while exploring clinical and laboratory data in relation to the study groups.

### Conclusions

Inflammatory and coagulation system activation of patients during ECMO support remains a major contributor to ECMO-related morbidity and mortality. NO may be an effective treatment for children supported on ECMO to mitigate inflammation and coagulation system activation. Data from the NECTAR pilot study will inform the design of a larger, definitive trial.

## Abbreviations

APTT: activated partial thromboplastin time
DSMB: data and safety monitoring board
ECMO: extracorporeal membrane oxygenation
HREC: Human Research and Ethics Committee
IL: interleukin
INR: international normalized ratio
MetHb: methemoglobin
NECTAR: Nitric Oxide on Extracorporeal Membrane Oxygenation in Neonates and Children
NO: nitric oxide
PICU: pediatric intensive care unit
PT: prothrombin time
RCT: randomized controlled trial
TEG: thromboelastography
TNF-*α*: tumor necrosis factor *α*
VA: veno-arterial
VV: veno-venous

## Data Availability

Deidentified participant data will be shared upon reasonable request after the publication of the original paper. Data will be available for use if the request is approved by the appropriate institutional review board and the Trial Steering Committee and a signed data access agreement is in place.
